# Radiation-Associated Sarcoma of the Breast in a Patient With a Germline Tumor Protein p53 Mutation

**DOI:** 10.7759/cureus.18563

**Published:** 2021-10-07

**Authors:** Cheyenne Thompson, Muhammad O Hakim, Jorge Infante-Mendez, Susan Kesmodel, Neha Goel

**Affiliations:** 1 Surgical Oncology, University of Miami Sylvester Comprehensive Cancer Center, Miami, USA; 2 Surgical Oncology, University of Miami Hospital, Miami, USA; 3 Pathology, University of Miami Hospital, Miami, USA; 4 Radiology, University of Miami Hospital, Miami, USA

**Keywords:** myofibroblastic sarcoma, adjuvant radiation therapy, li-fraumeni syndrome, germline tp53 mutation, radiation-induced sarcoma

## Abstract

Radiation-induced sarcoma of the breast is a rare complication that is primarily treated with surgical resection but in patients with advanced disease, a multimodality treatment approach is often required. This case report discusses a 37-year-old female with a history of a pT3N3M0, estrogen receptor (ER)+, progesterone receptor (PR)+, human epidermal growth factor receptor 2 (HER2)+, right breast cancer, and a germline tumor protein (TP) p53 mutation who underwent right modified radical mastectomy, adjuvant systemic therapy, and radiation therapy, and subsequently developed a radiation-induced sarcoma.

The patient is a 37-year-old female who has a history of pT3N3M0, ER/PR+, HER2+, and right breast cancer diagnosed in 2014. At the time of diagnosis, she had locally advanced disease and underwent right modified radical mastectomy followed by adjuvant chemotherapy, radiation, delayed right breast implant-based reconstruction, and left breast augmentation with mastopexy. Upon completion of adjuvant chemotherapy, she was started on hormonal therapy. In February 2020, she underwent genetic testing given her early onset of breast cancer and was found to have a germline TP53 mutation. Routine MRI for breast implant evaluation showed two irregular enhancing masses with an additional satellite lesion in the right breast. Right breast ultrasound (US)-guided biopsy revealed two separate foci of high-grade pleomorphic fibroblastic/myofibroblastic sarcoma. Further staging workup with a whole-body MRI was negative for evidence of metastatic disease. Her case was discussed in multidisciplinary sarcoma tumor board and consensus was for surgical resection. She underwent radical resection of the right chest wall masses and subcutaneous tissue, removal of right breast implant and capsulectomy, and left breast mastectomy with left breast implant removal and capsulectomy. The final pathology revealed two separate foci of high-grade pleomorphic fibroblastic/myofibroblastic sarcoma, 1.2 cm and 1.1 cm in their greatest dimensions with negative margins. Her case was re-discussed in multidisciplinary sarcoma tumor board and due to T1 size of the tumors and the negative resection margins, close surveillance with annual whole-body MRI and quarterly chest MRI imaging was recommended.

In patients with a germline TP53 mutation and breast cancer, the utilization of adjuvant radiotherapy should be considered cautiously given the increased risk of radiation-associated sarcoma.

## Introduction

Radiation-associated sarcoma of the breast is a rare complication of adjuvant radiation therapy for cancer with an incidence of less than 0.05% [1]. However, in patients with germline mutations, there is an elevated risk of radiation-associated tumors. In a series of sarcomas developing in the irradiation field in patients previously treated with radiotherapy, Gonin-Laurent and colleagues found a high rate of TP53 mutations (58%) suggesting this gene plays a role in the formation of the sarcomas [2]. This disease is primarily treated with surgical resection but in patients with advanced disease, a multimodality treatment approach is often required. This case report discusses a young female with a history of locally advanced right breast cancer who underwent right modified radical mastectomy, adjuvant systemic therapy, and radiation therapy and subsequently developed a sarcoma inside the radiation field.

## Case presentation

The patient is a 37-year-old female who has a history of right-sided breast cancer diagnosed in 2014. At the time of diagnosis, she had locally advanced disease (cT3N3M0, ER+, PR+, HER2+). She underwent a right modified radical mastectomy and final pathology revealed pT3N3M0 disease. She received adjuvant systemic therapy with doxorubicin and cyclophosphamide followed by Taxol and Herceptin. She then received adjuvant radiation therapy to the chest wall and regional lymph nodes. Upon completion of systemic therapy and radiation, she was started on tamoxifen and Zoladex in November 2015. In February 2017, she underwent right breast reconstruction with a silicone implant and left breast augmentation mastopexy.

**Figure 1:**
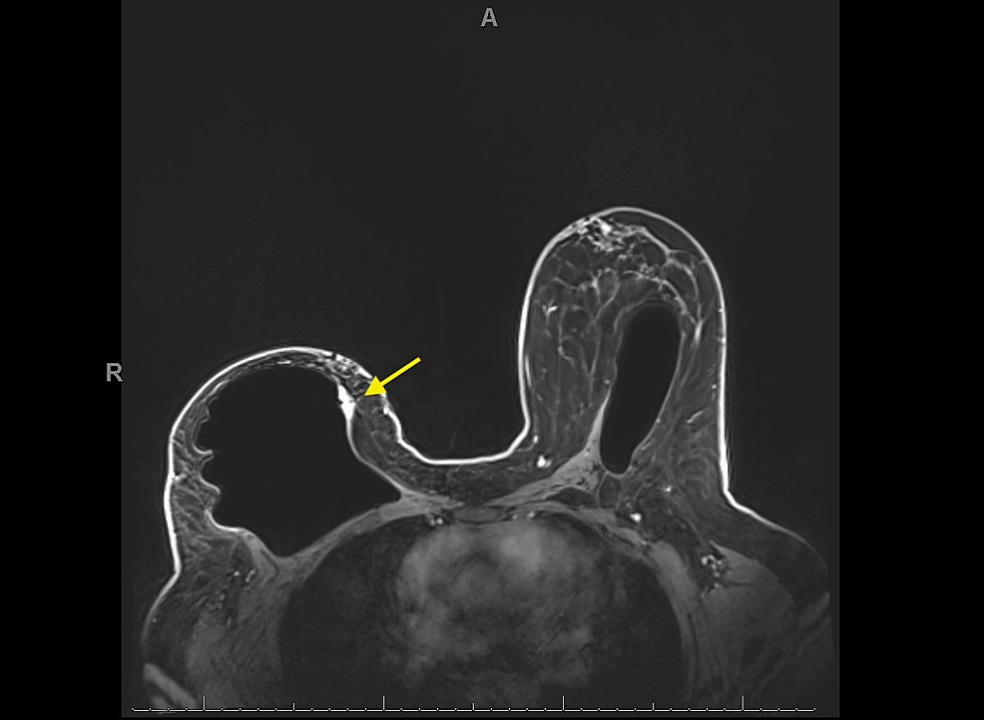
Bilateral breast MRI Right breast irregular enhancing mass (yellow arrow) at the 3:00 axis next to the implant measuring 1.9 cm anterior-posterior by 0.8 cm transverse by 0.9 cm craniocaudal located 2.7 cm from the nipple.

**Figure 2:**
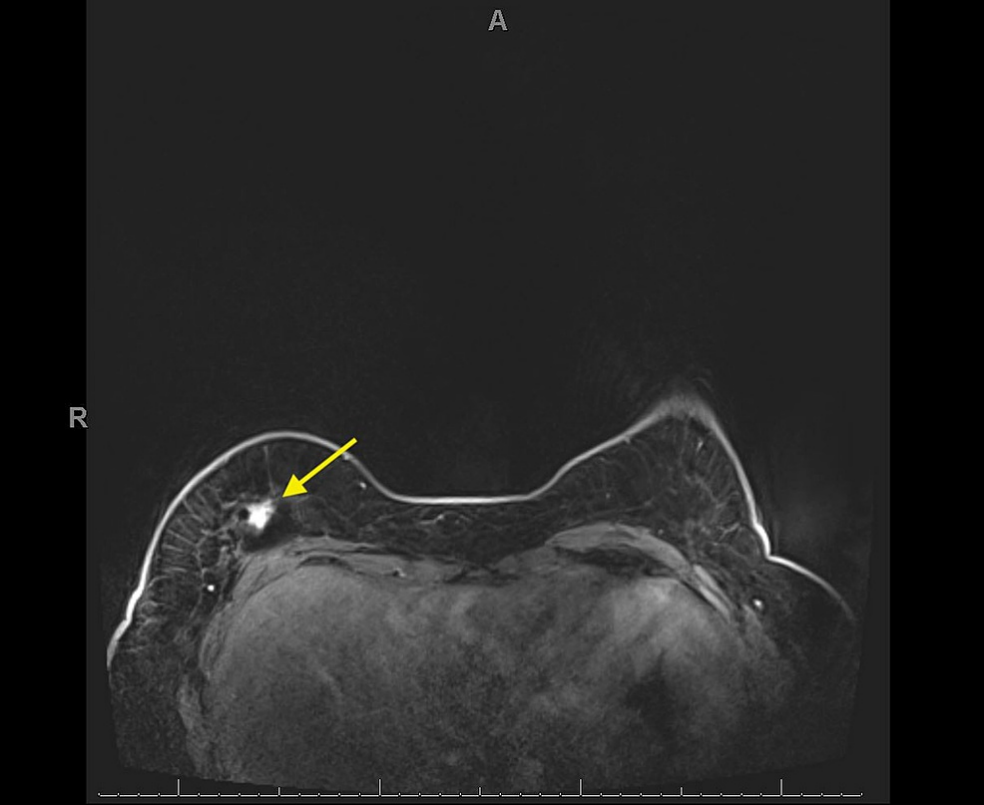
Bilateral breast MRI Right breast irregular enhancing mass (yellow arrow) at the 6:00 axis posteriorly measuring 2.9 cm anterior-posterior by 2.1 cm transverse by 0.9 cm craniocaudal located 8.4 cm from the nipple.

In February 2020, she established care with a different institution and underwent genetic testing given early onset of her breast cancer. Results showed a germline TP53c.742C>T mutation and variants of uncertain significance in MSH2 and PMS2. A routine six-month follow-up MRI for her implant performed in February 2020 showed two new right breast irregular enhancing masses at three o’clock (Figure 1) and six o’clock (Figure 2). Right breast US-guided biopsy in March 2020 revealed two separate foci of high-grade pleomorphic fibroblastic/myofibroblastic sarcoma at the three o’clock and six o’clock positions. Immunohistochemistry showed that the neoplastic cells were positive for p53 and rare cells were positive for desmin. The patient underwent additional cross-sectional MRI imaging of the chest/abdomen/pelvis which showed no evidence of metastatic disease. The case was reviewed in multidisciplinary sarcoma tumor board and due to the small size of the tumors (T1) and since the patient was an operative candidate, the recommendation was to proceed with surgery first.

**Figure 3:**
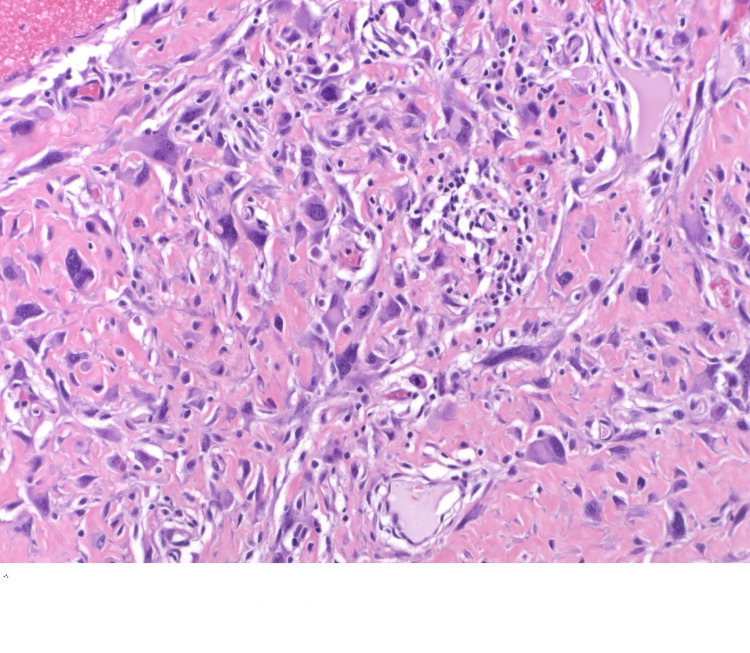
Fibroblastic sarcoma that developed in the radiation field Pleomorphic spindle cells arranged in intersecting fascicles and enmeshed in a collagenous stroma.

**Figure 4:**
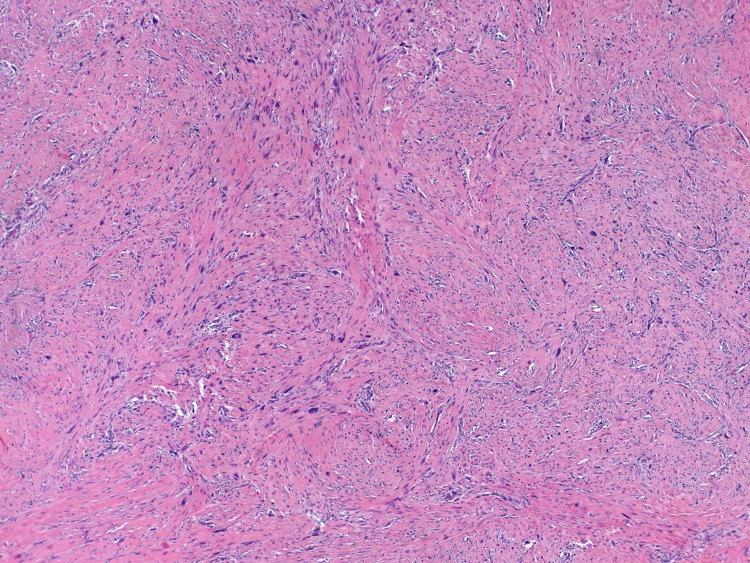
Fibroblastic sarcoma that developed in the radiation field The tumor cells have large nuclei irregular nuclei and intimately related to the collagenous stroma that also contains scattered chronic inflammatory cells.

On May 4, 2020, she underwent radical resection of the right chest wall skin and subcutaneous tissue with removal of the right breast implant and capsulectomy. Due to the high risk of breast cancer associated with Li-Fraumeni Syndrome, left breast contralateral prophylactic mastectomy with left breast implant removal and capsulectomy was also performed. Concurrently, she underwent tissue re-arrangement with a local advancement flap by plastic surgery for closure of the right chest wall. The final pathology from the right chest wall resection revealed two separate foci of high-grade pleomorphic fibroblastic/myofibroblastic sarcoma (Figures 3, 4), 1.2 cm and 1.1 cm in greatest dimension, with an R0 resection. Her case was discussed in multidisciplinary sarcoma tumor board postoperatively and due to the small size of the tumors (T1) and the negative resection margins, no adjuvant therapy was recommended. She will undergo annual whole-body MRI and three-month chest MRI imaging for surveillance.

## Discussion

Although radiation therapy is an integral treatment modality for multidisciplinary cancer treatment, exposure to this ionizing radiation increases an individual’s risk of developing a secondary malignancy, particularly soft tissue sarcoma, with a strong dose-response relationship [3]. These iatrogenic tumors arise within the field of therapeutic radiation following a latency period and are not recurrences of the original cancer. Among patients without genetic mutations, radiation-associated sarcoma is rare with a cumulative incidence that increases with time. In a retrospective study by Kirova et al., the incidence was calculated to be 0.9% at 15 years in patients treated with radiation therapy compared with 0.1% in patients who did not receive radiation therapy [4]. Petry et al. noted that the incidence of radiation-induced malignancies in patients with TP53 mutations who received adjuvant radiation therapy was 16.6% [5]. There are a variety of histologic subtypes associated with radiation-induced sarcomas and they include unclassified pleomorphic sarcoma, angiosarcoma, malignant peripheral nerve sheath tumors, and leiomyosarcoma [6]. For patients presenting without metastasis, the five-year overall survival rate is 44% [7].

Due to their rare occurrence, the molecular characterization of radiation-induced malignancy is limited although it has been found that the TP53 gene plays an early role in radiation-induced carcinogenesis [2,8]. The major feature of the TP53 mutations in radiation-induced sarcomas is a high rate of deletions (with an excess of deletions when compared to relative insertions) attributed to DNA double-strand breaks which result from ionizing radiation in cells [2,3]. Ionizing radiation directly damages DNA and can generate a variety of lesions and in multiple locations [3]. These breaks in DNA can be a direct consequence of energy deposits along the radiation track or an indirect consequence during repair of damaged sites [2]. Targeted gene screens have been performed on radiation-induced sarcomas and have shown an increase in the amount of deletions and substitutions along with frequent inactivation of the TP53 and RB1 genes [3].

Diagnostic criteria for radiation-associated sarcoma require that the lesion be located within the irradiated region and that the pathology differs from that of the original cancer. Additionally, a latency period should have elapsed with the median time between radiation therapy and a diagnosis of sarcoma being 10 years [7]. Factors such as patient age at exposure to radiation, radiation dose, and susceptibility to specific tissue types of radiation-induced cancers influence an individual’s risk for developing radiation-associated sarcoma. Tissues that are known to have a higher susceptibility to radiation-induced malignancy include bone marrow, female breast, and thyroid tissues [9]. There is an overall poor prognosis associated with radiation-induced sarcoma as the treatment for most patients is late and ineffective. Due to the late presentation of this disease, careful long-term follow-up is necessary for early detection. The standard treatment is radical surgical resection with wide margins [4]. As a result, this often requires staged reconstruction with a plastic surgeon once margins are confirmed to be negative. As a component of the treatment plan, systemic chemotherapy and re-radiation are considered given the overall poor prognosis, but the use of radiation therapy should be weighed with caution, particularly in patients with a TP53 mutation [6].

Given the potential detrimental effects of ionizing radiation on patients with impaired p53 function, methods of surveillance and screening in patients with Li-Fraumeni Syndrome should be cautiously considered. Li-Fraumeni Syndrome patients are advised to avoid radiation exposure due to the risk of developing radiation-induced tumors [10]. CT utilizes specified doses of radiation to acquire images and can be augmented with the use of contrast agents. MRI is another modality of imaging that does not utilize radiation or contrast agents to acquire high-definition images. This modality utilizes and manipulates the magnetism of protons to detect and reconstruct an image [11]. Whole-body MRI can serve as an effective screening and surveillance tool in patients with Li-Fraumeni Syndrome or other cancer-predisposing syndromes [10].

When treating breast cancer in a patient with Li-Fraumeni Syndrome, the risk of developing contralateral breast cancer should also be considered. Especially those diagnosed at an early age, contralateral breast cancer rates are substantial in TP53, Breast Cancer Gene 1 (BRCA1), and BRCA2 mutation carriers. A study by Hyder and colleagues found that the contralateral breast cancer rate in those without a germline mutation was 0.6 per 1000 while the rate in mutation carriers was approximately 2 per 1000 [12]. Given this increased risk, the option of risk-reducing mastectomy should be discussed with the patient when determining the treatment plan.

## Conclusions

Radiation-induced sarcoma of the breast is a rare complication of adjuvant radiation therapy however, there is an elevated risk for patients with TP53 mutations. While surgical resection is the primary treatment modality for patients with operable disease, because of the aggressive nature of these tumors, a multimodality treatment approach is required. This patient presented with a locally advanced breast cancer and was treated with adjuvant radiation therapy at an outside institution before it was known that she had a TP53 mutation. Her radiation-associated sarcoma presented at an early, localized stage, and was managed with surgery alone. However, in patients with TP53 mutations and breast cancer, treatment approaches that avoid adjuvant radiation therapy and surveillance which reduces use of computerized tomography radiation should be considered to reduce the risk of radiation-associated sarcoma.
